# Supplementary material for: Lactation opposes pappalysin‐1‐driven pregnancy‐associated breast cancer
Source: EMBO Mol Med. 2016 Mar 8;8(4):388–406. doi: 10.15252/emmm.201606273 (PMC4818749; doi:10.15252/emmm.201606273)

Appendix

Table of contents:

Appendix figure legends S1 – S8
Appendix figures S1-S8

Appendix Figure S1: Expression levels of PAPP-A in non-transgenic and transgenic mice and magnification/inset of involution time course whole mount.

A. Representative (from n=3 mice) histological sections of mammary glands from 6-week old female mice, nontransgenic (NT) vs PAPP-A transgenic (PAPP-A). Left: H&E. Right: PAPP-A immunohistochemistry, scale bar: 50μm.
B. Representative (from n=12 mice, 3 mice/time point) NT vs. PAPP-A mammary gland whole mount, involution time course (preceded by a 2-day lactation), Scale bar, 2mm. Boxed regions correspond to area imaged in Figure 1.
C. Representative (from n=12 mice, 3 mice/time point) involution time course (preceded by a 2 week lactation), Scale bar, 2mm. Boxed regions correspond to area imaged in Figure 4.

Appendix Figure S2: Bar graph representation of data from Figures 2B, 2D and 5D.

A. Quantification values from Figure 2B of Nontransgenic (NT) vs. PAPP-A Transgenic (PAPP-A) involuting mammary glands. (Average of 3 determinations/mouse, n=3 mice/time point). Bars represent the mean ± SD. Unpaired t-test (two-tailed): *p(day6)=0.0408, *p(day12)=0.0440.
B. Quantification values from Figure 2D of NT vs. PAPP-A involuting mammary glands. (Average of 3 determinations/mouse, n=3 mice/time point). Bars represent the mean ± SD. Unpaired t-test (two-tailed): **p(day6)=0.0095,*p(day12)=0.0164.
C. Quantification values of collagen by Masson's trichrome stain from Figure 5D of NT vs. PAPP-A pregnancy mammary glands. (Average of 3 determinations/mouse, n=3 mice/time point). Bars represent the mean ± SD. Unpaired t-test (two-tailed): ****p(day9)<0.0001, **p(day12)=0.0032.

Appendix Figure S3: Analysis of collagen levels by RT-PCR and circularly polarized light microscopy.

A. Median mouse col1a transcript levels (with range) from involuting mammary glands of NT and PAPP-A mice. (n=3 mice/time point, triplicate experiment). Bars represent the mean±SD. Unpaired t-test (two-tailed): ***p=0.00007, **p=0.0100.
B. Circularly polarized light microscopy of Picrosirius red stained mammary gland slides
C. Red channel (thick collagen bundles) of images from B.

Appendix Figure S4: Control proteolytic assay of recombination rIGFBP-5 in presence of laminin.

A. Proteolysis assay of recombinant IGFBP-5 (rIGFBP-5) incubated with culture media from MCF7 control (ctl) vs. PAPP-A (p-a) cells, in the presence of conditioned media (CM), laminin, or collagen.
B. Quantification of Western Blot from S4A.

Appendix Figure S5: Individual quantification values from xenograft data of Figures 3F, H, I, and J.

A. Individual quantification values from (3F) of matrigel/collagen xenograft tumor volume of MCF-7 control (ctl) or PAPP-A (p-a) cells.(Average of 2 tumors/mouse, 8 tumors total, n=4 mice/group). Bars represent the mean ± SD. Unpaired t-test (two-tailed): ***p=0.0008.
B. Individual quantification values from (3H) of virgin (-V) xenograft tumor volume of ctl or p-a cells. (Average of 2 tumors/mouse, 8 tumors total, n=4 mice/group). Bars represent the mean ± SD. Unpaired t-test (two-tailed): p=0.0811.
C. Individual quantification values from (3I) of involution (-I) xenograft tumor volume of ctl or p-a cells.(Average of 2 tumors/mouse, 8 tumors total, n=4 mice/group). Bars represent the mean ± SD. Unpaired t-test (two-tailed): **p=0.0092.
D. Individual quantification values from (3J) of involution (-I) xenograft growth rate (slope of graph 2J) of ctl or p-a cells. (Average of 1-2 tumors/mouse, 5 tumors total, n=3 mice/group). Bars represent the mean ± SD. Unpaired t-test (two-tailed): **p=0.0031.

Appendix Figure S6: Individual quantification values from xenograft data of Figures 5B-D.

A. Individual quantification values from (6B) of involution (-Inv.) xenograft tumor volume of MCF-7 control (ctl) or PAPP-A (p-a) cells. (Average of 2 tumors/mouse, 6 tumors total, n=3 mice/group). Bars represent the mean ± SD. Unpaired t-test (two-tailed): **p=0.0027.
B. Individual quantification values from (6C) of lactation (-Lac.) xenograft tumor volume of ctl or p-a cells. (Average of 2 tumors/mouse, 6 tumors total, n=3 mice/group). Bars represent the mean ± SD. Unpaired t-test (two-tailed): p=0.4359.
C. Individual quantification values from (6D) of lactation + involution (-Lac.+Inv.) xenograft tumor volume of ctl or p-a cells. (Average of 2 tumors/mouse, 6 tumors total, n=3 mice/group). Bars represent the mean ± SD. Unpaired t-test (two-tailed): p=0.3066.

Appendix Figure S7: Transcript levels of STC1 and STC2, IGFBP-5 protein levels, and immunodepletion of STC1 and STC2 in mouse mammary gland at various stages of mammary development.

A. mouse STC1 (left) and STC2 (right) transcript levels from whole mouse mammary gland of virgin (V), pregnancy day 12 (P), lactation day 12 (L), and involution day 12 (I) wild type females. Each bar represents the mean ± SD of three independent experiments. One-way ANOVA: all p>0.05.
B. Pregnancy (top) or lactation (bottom) extracts were immunodepleted with antibodies shown and assayed for rIGFBP-5 degradation.
C. Pregnancy (left) or lactation (right) extracts immunodepleted with STC1, STC2, or STC1/STC2 were assessed for PAPP-A levels by ELISA. Positive control: anti-PAPP-A, negative control: goat IgG. Immunodepleted supernatant. Each bar represents the mean ± SEM of three independent experiments. One-way ANOVA with Tukey's post-hoc test: goat IgG ***p=0.0001 for pregnancy, **p=0.0040 for lactation.

D. Relative IGFBP-5 levels in a non-transgenic mouse mammary gland at indicated phases and density quantification. Mean ± SD of three independent experiments.

Appendix Figure S8: Summary of tumor characteristics and TACS-3 score variations in the parous and nulliparous breast cancer patients.

A. Table of tumor size and grade of parous and nulliparous patients. Unpaired t-test (alpha=0.05) was performed; There was no significant difference between the two groups.
B. Intratumor variation in TACS-3 score from Figure 7F, as represented by the mean±SEM of 6 region of interests (ROI) analyzed per patient sample.

Appendix Figure S1

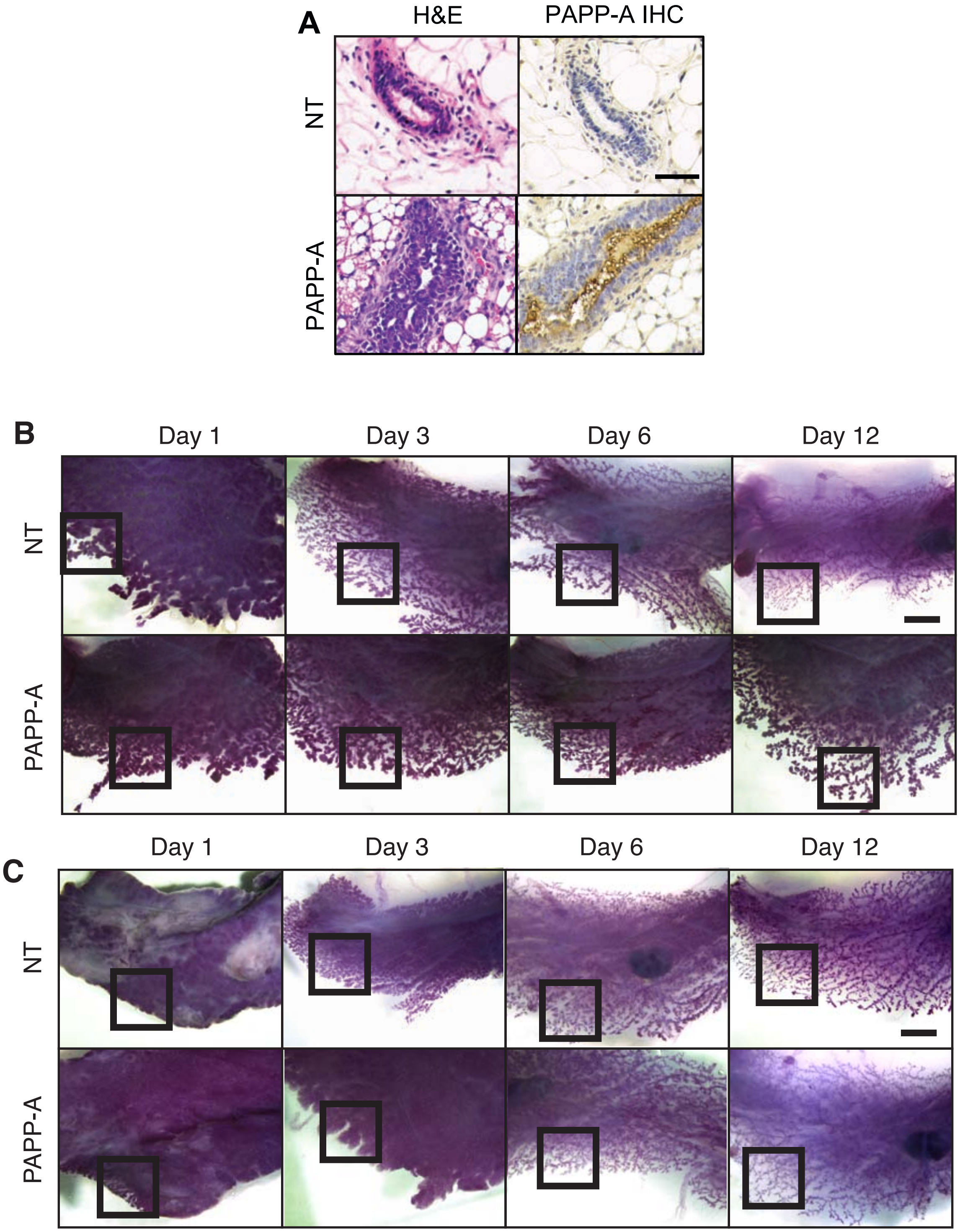

Appendix Figure S2

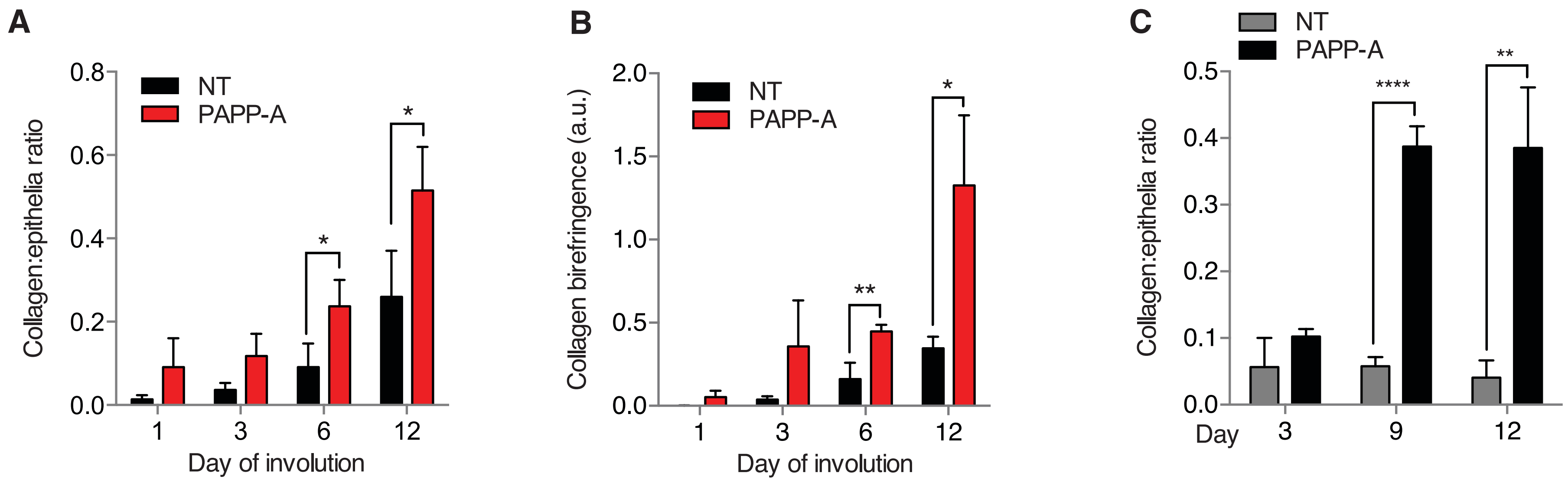

Appendix Figure S3

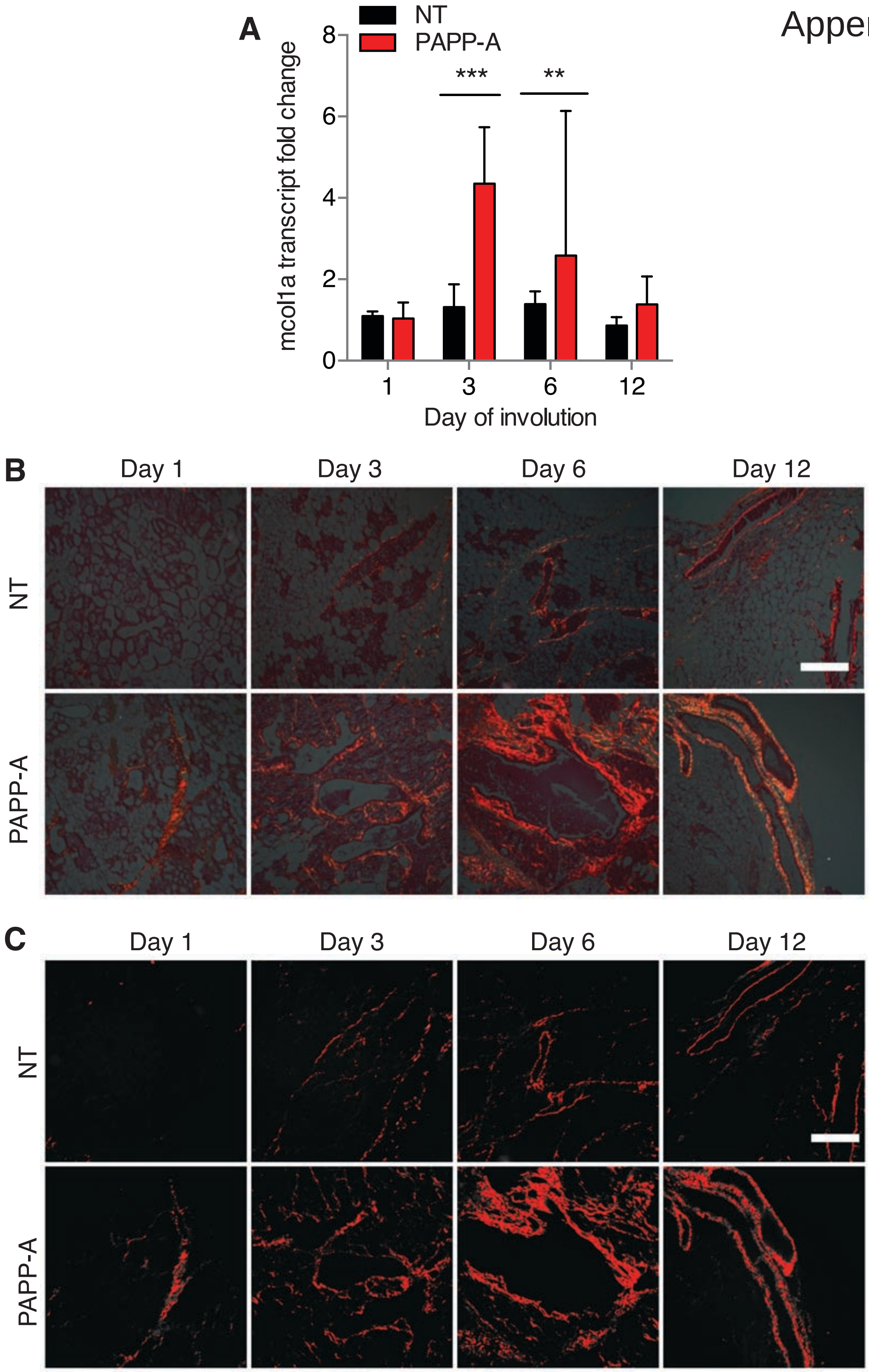

Appendix Figure S4

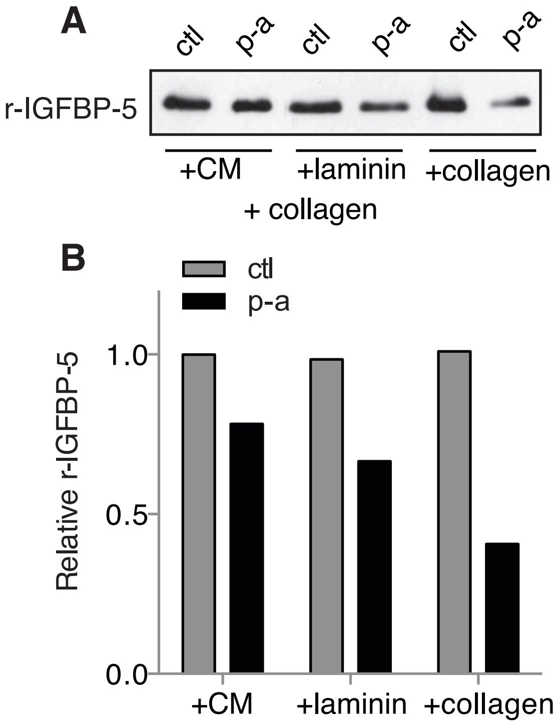

Appendix Figure S5

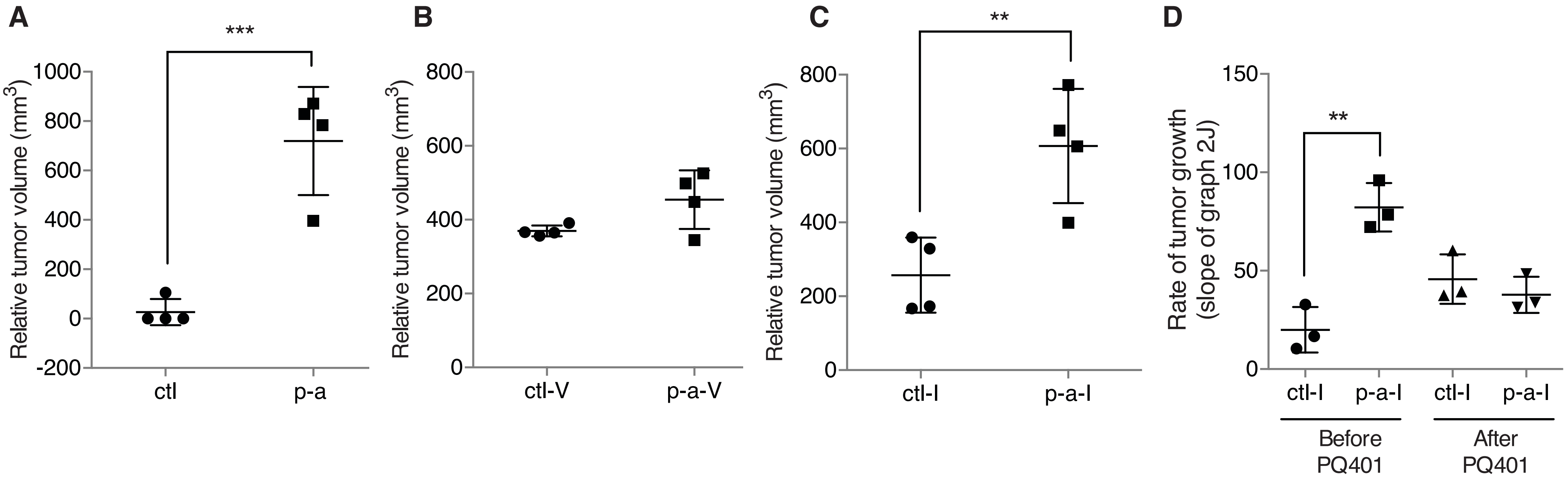

Appendix Figure S6

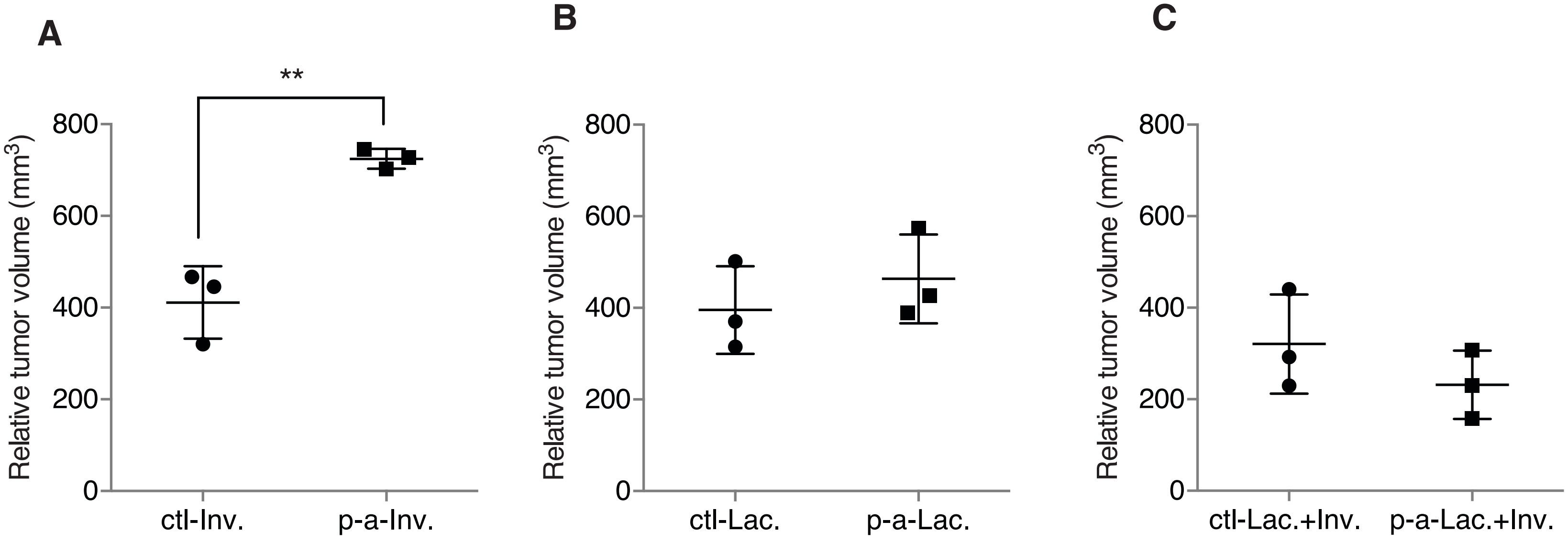

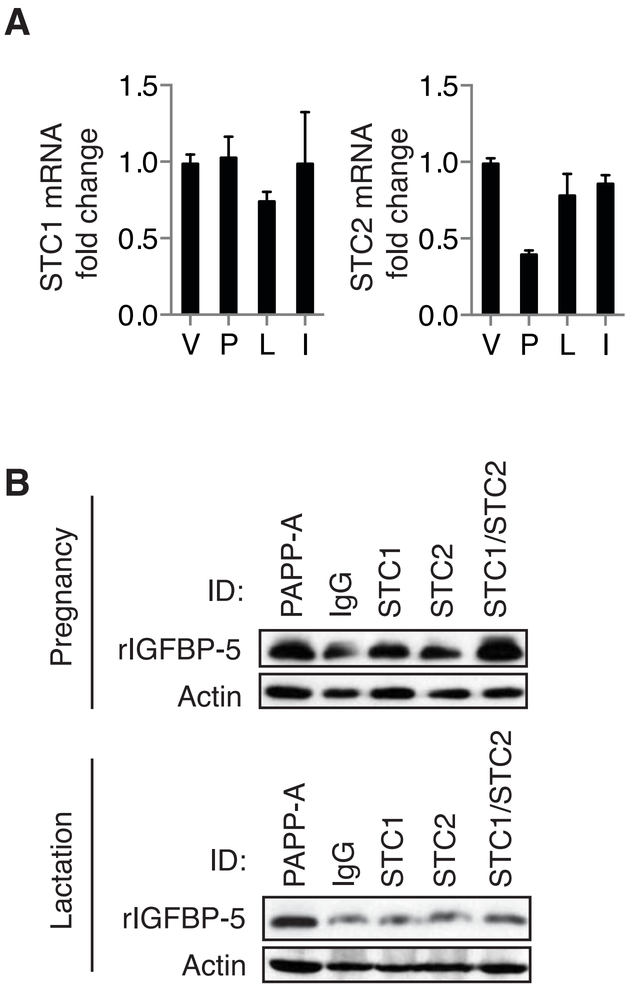

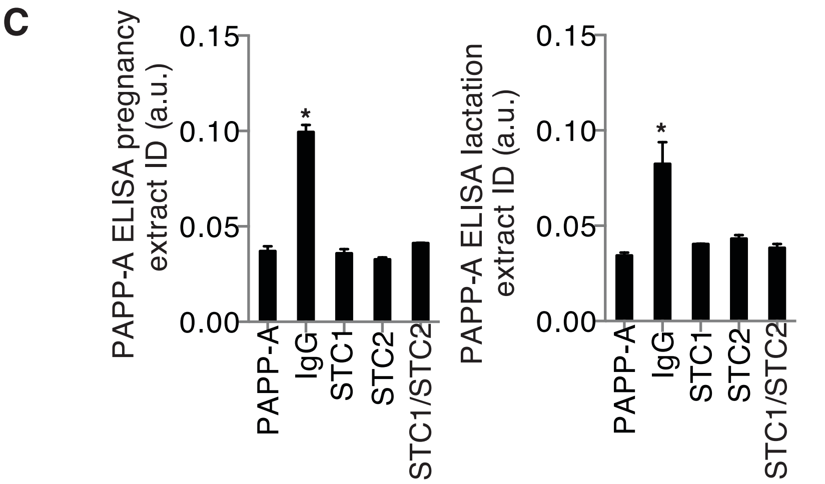

D

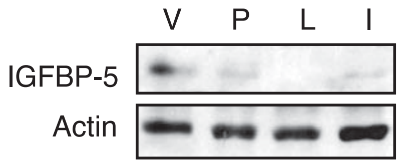

Relative IGFBP-5 levels	mean	S.D.
Virgin	1.000	0.000
Pregnancy	0.639	0.270
Lactation	0.008	0.012
Involution	0.658	0.036

A

**Tumor size**	**Parous**	**Nulliparous**	*p-value*
Size in cm (mean±SEM)	1.876±0.334	1.532±0.271	0.463
Patient data available for:	n=19	n=13	

**Tumor Grade**	**Parous**	**Nulliparous**	*p-value*
DCIS (%)	4.167	0.000	0.757
I (%)	4.167	6.667	
II (%)	33.333	33.333	
III (%)	58.333	60.000	
Patient data available for:	n=24	n=15	

B

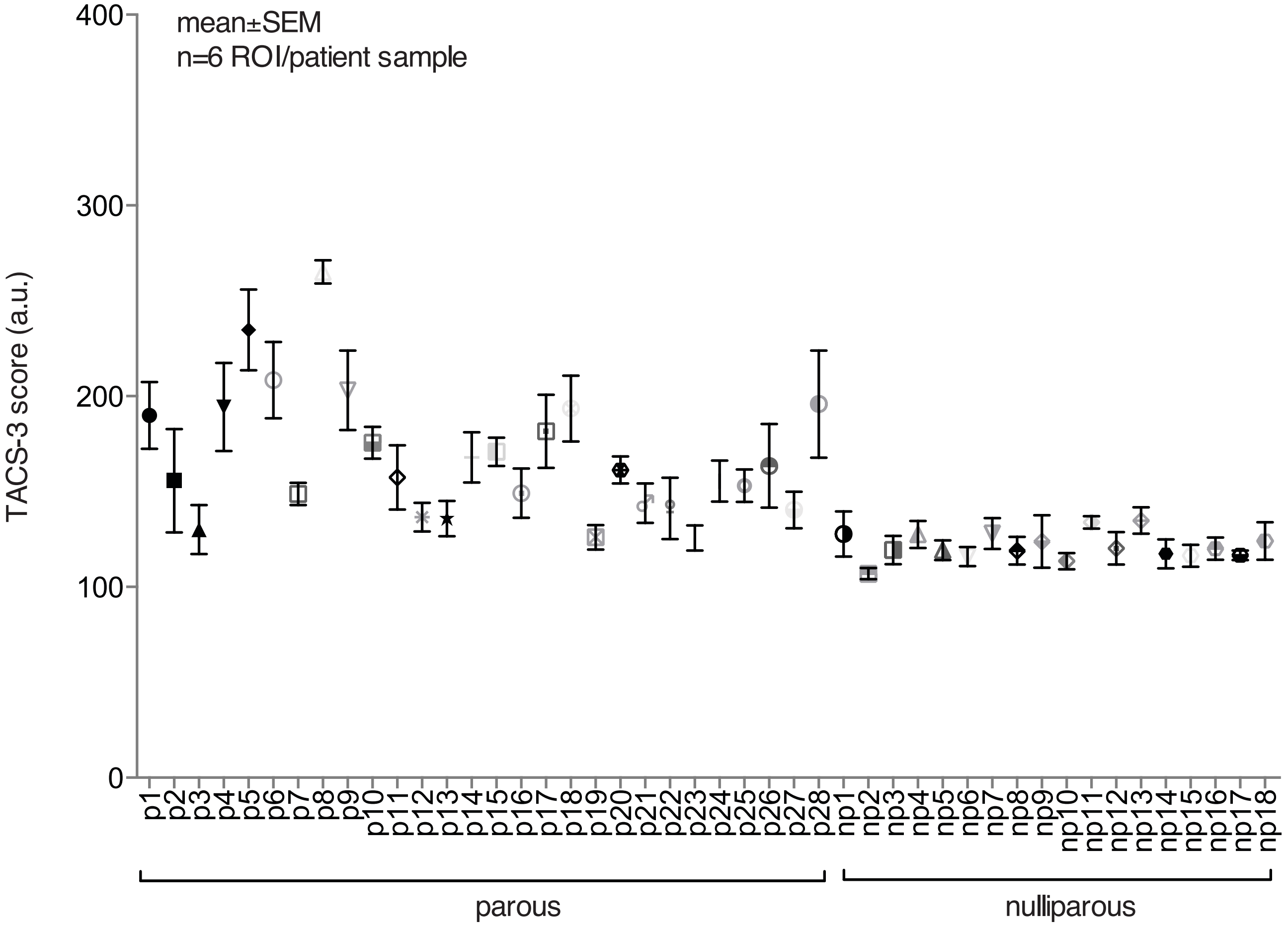

Supplement: Supplementary file 1 — Appendix [file EMMM-8-388-s001.pdf]
