# Supplementary figures and images for: Lactation opposes pappalysin‐1‐driven pregnancy‐associated breast cancer
Source: EMBO Mol Med. 2016 Mar 8;8(4):388–406. doi: 10.15252/emmm.201606273 (PMC4818749; doi:10.15252/emmm.201606273)

## Supplementary 4

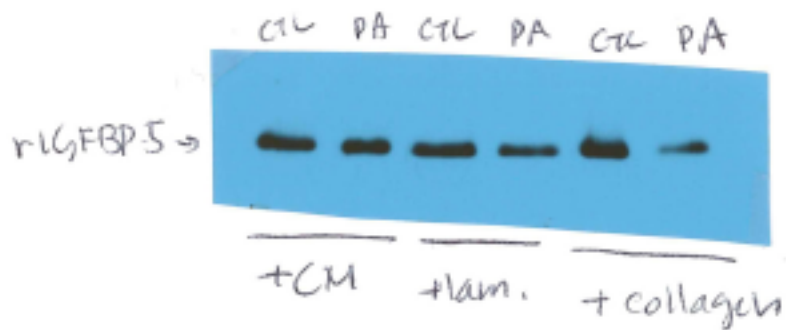

Supplement: Supplementary file 2 — Source Data for Appendix [file EMMM-8-388-s006.zip › Source data fig. S4.pdf]

Sup Fig. 7B

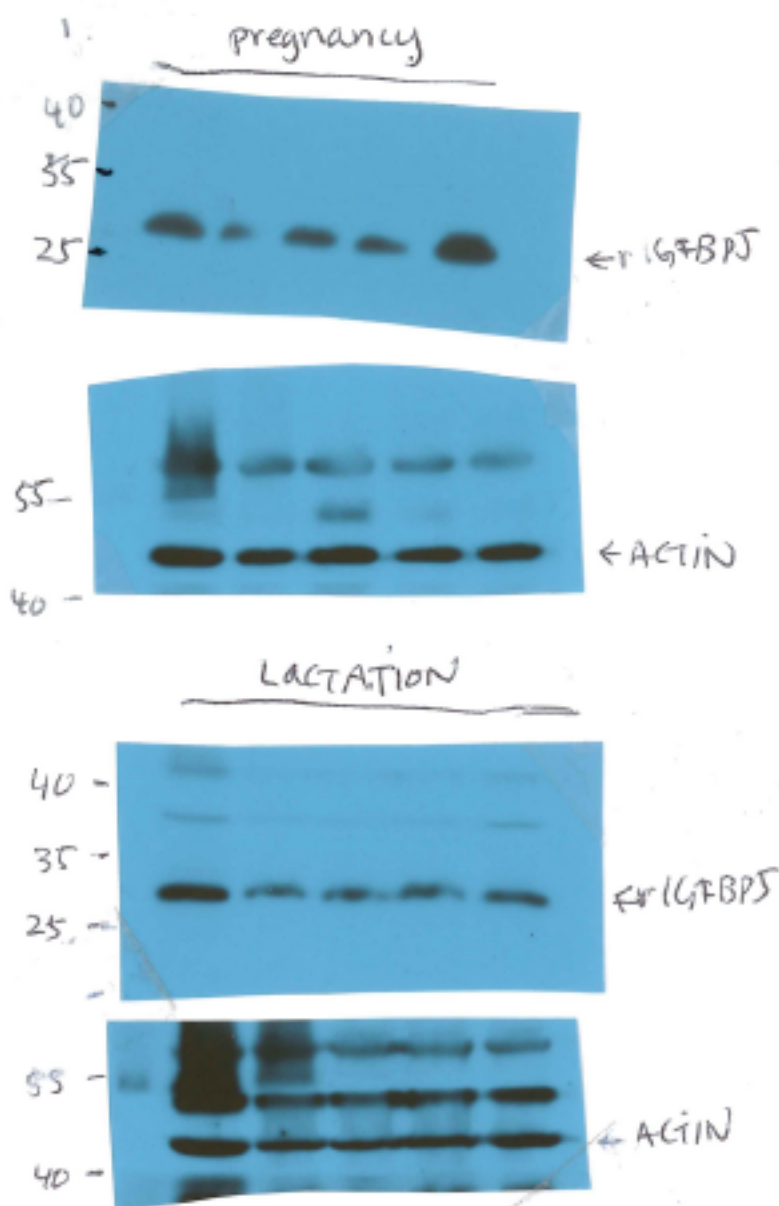

Sup Fig. 7D

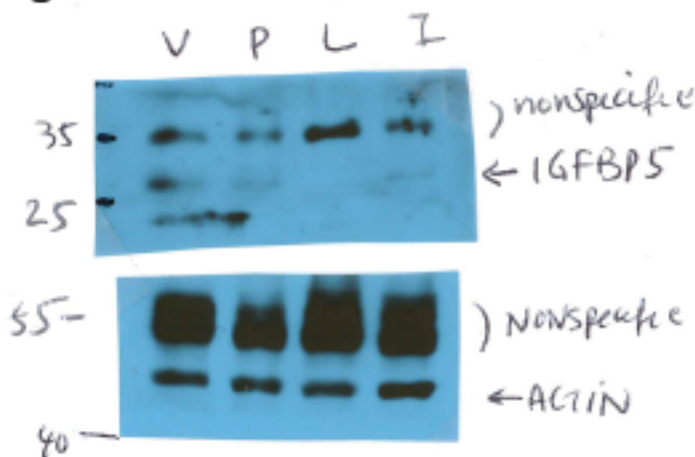

Supplement: Supplementary file 2 — Source Data for Appendix [file EMMM-8-388-s006.zip › Source data figure S7.pdf]

Figure 1

G

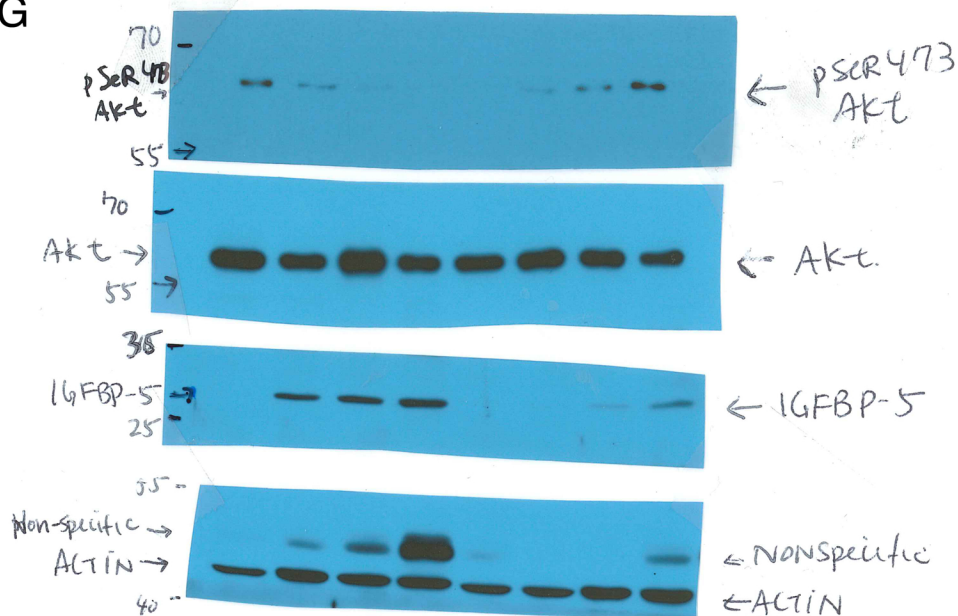

I

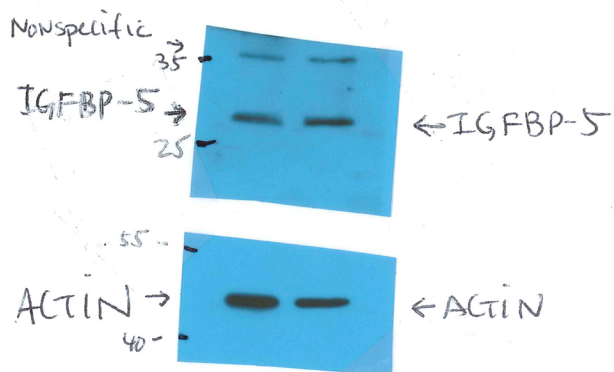

Supplement: Supplementary file 4 — Source Data for Figure 1 [file EMMM-8-388-s002.pdf]

Figure 3

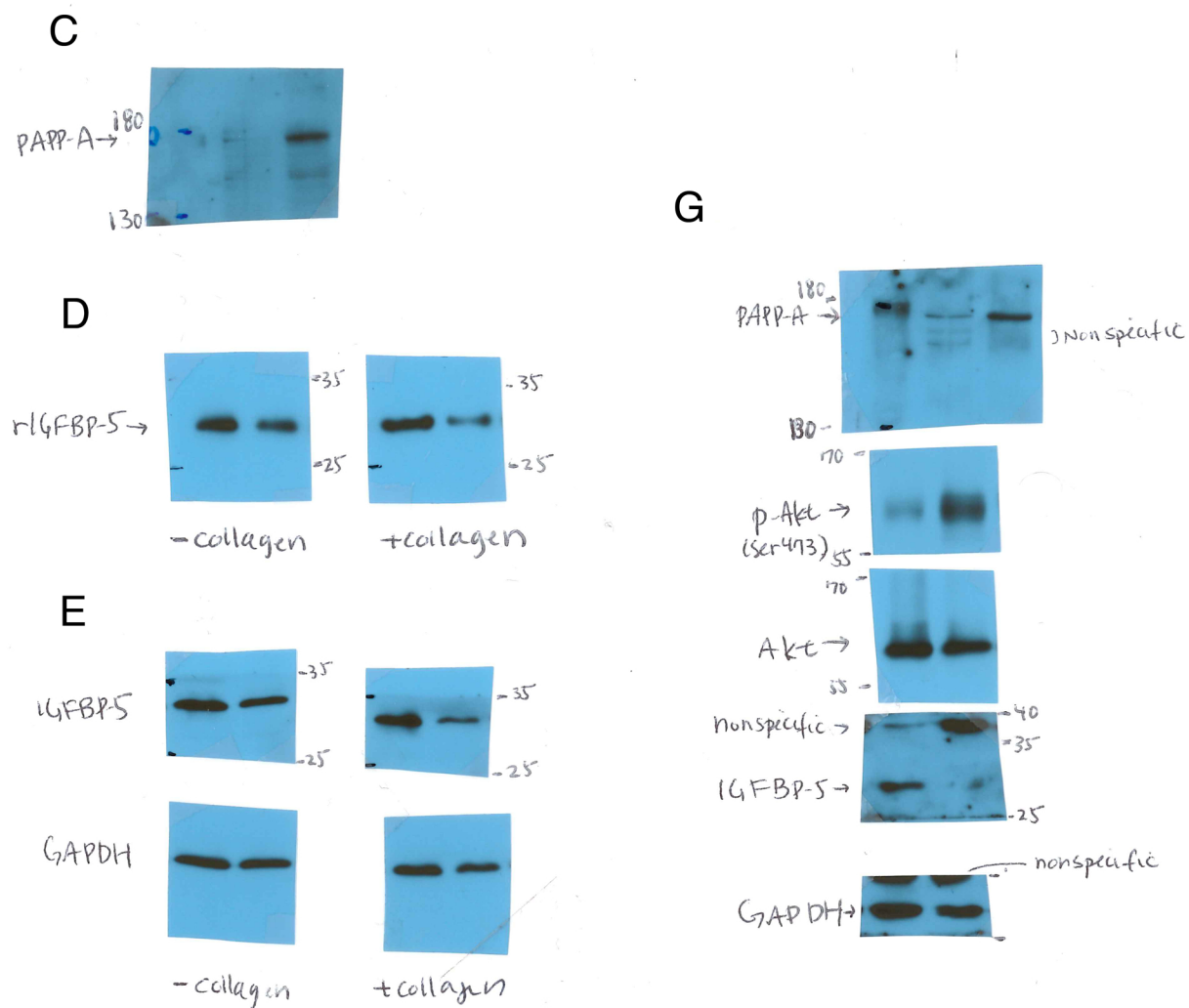

Supplementary 4

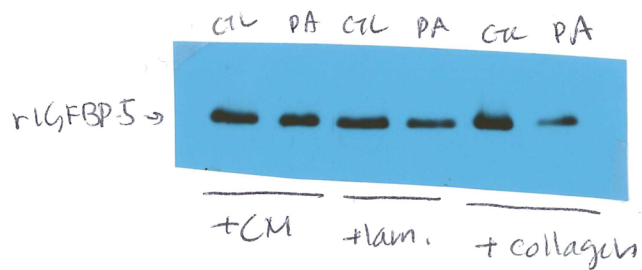

Supplement: Supplementary file 5 — Source Data for Figure 3 [file EMMM-8-388-s003.pdf]

Figure 5

A

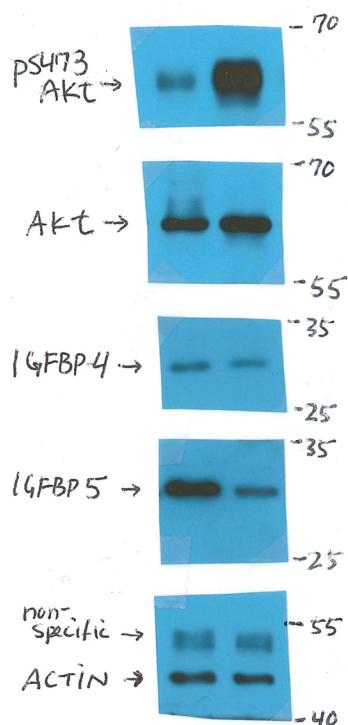

F

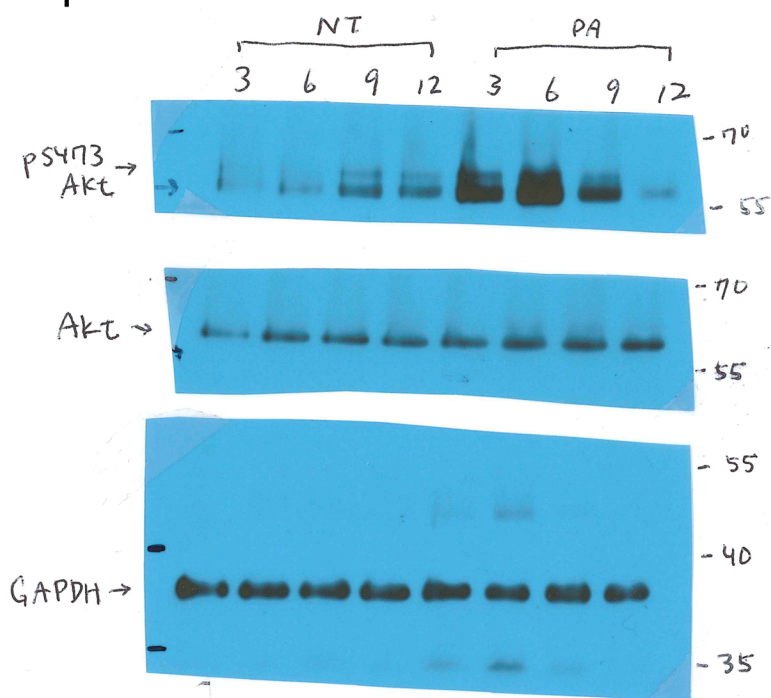

H

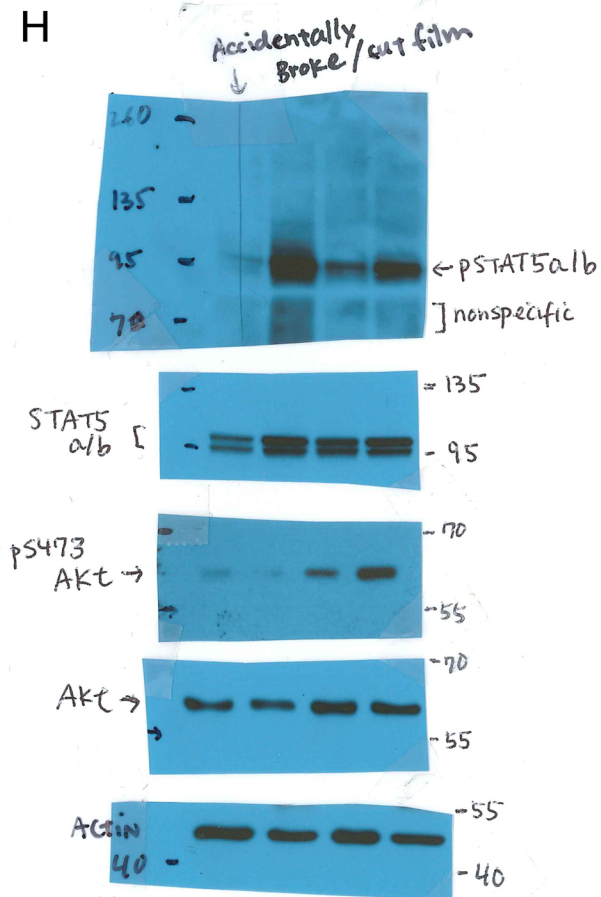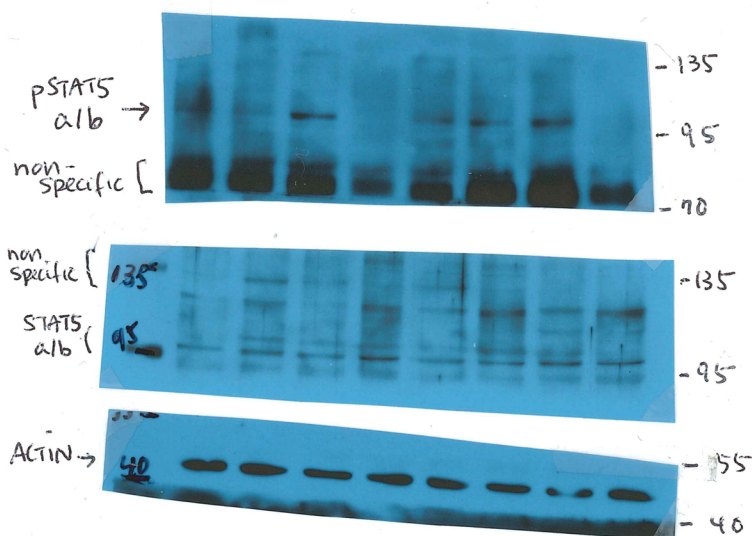

Supplement: Supplementary file 6 — Source Data for Figure 5 [file EMMM-8-388-s004.pdf]

Figure 6

G

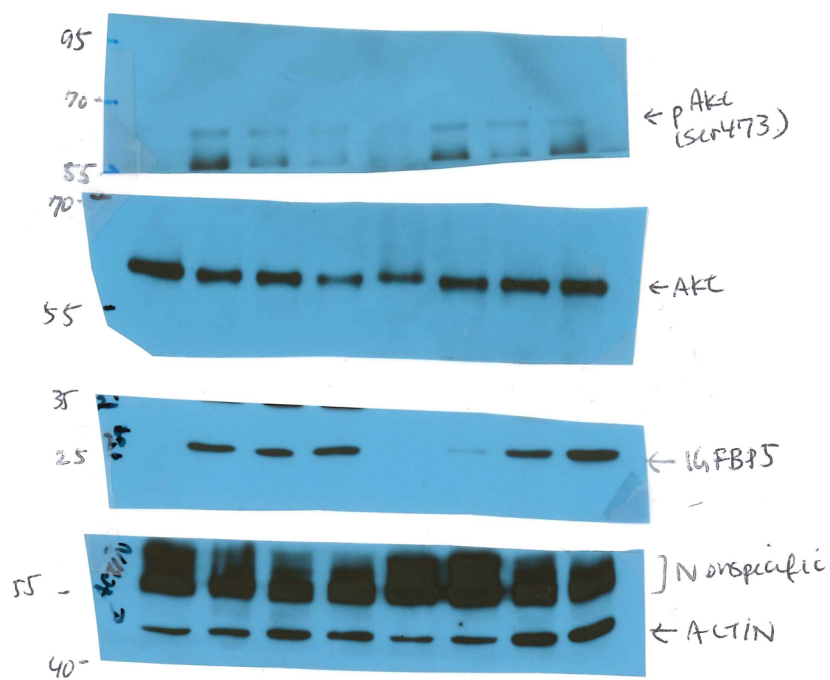

J

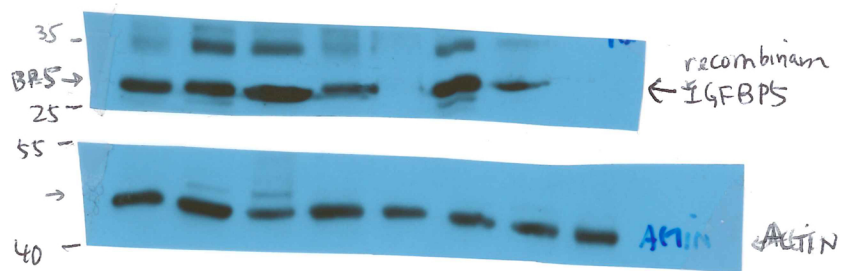

Sup Fig. 7B

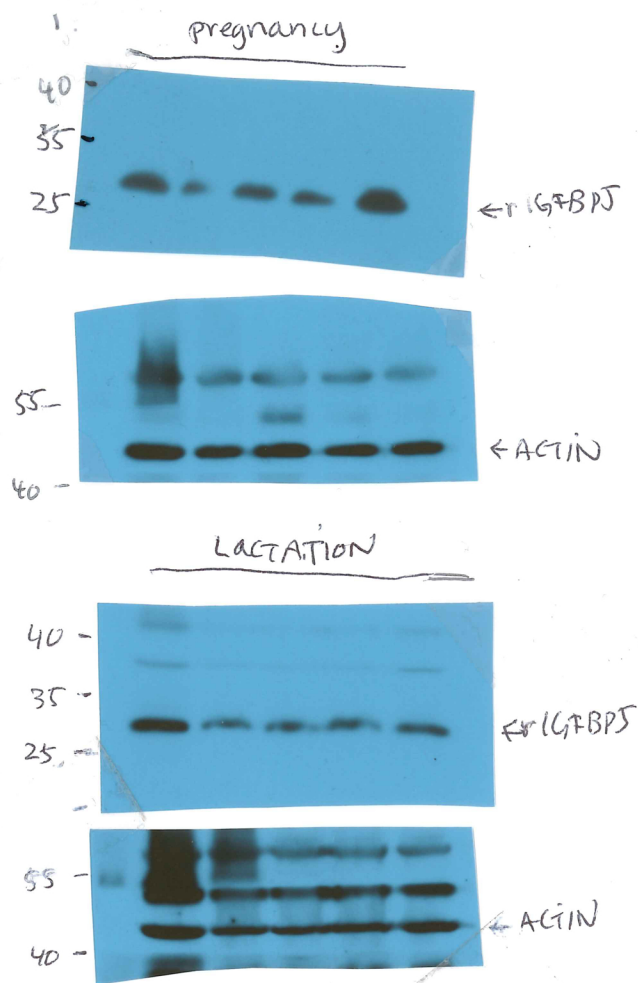

Sup Fig. 7D

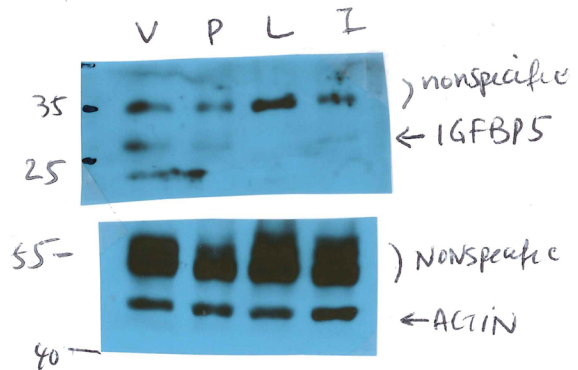

Figure 6

H

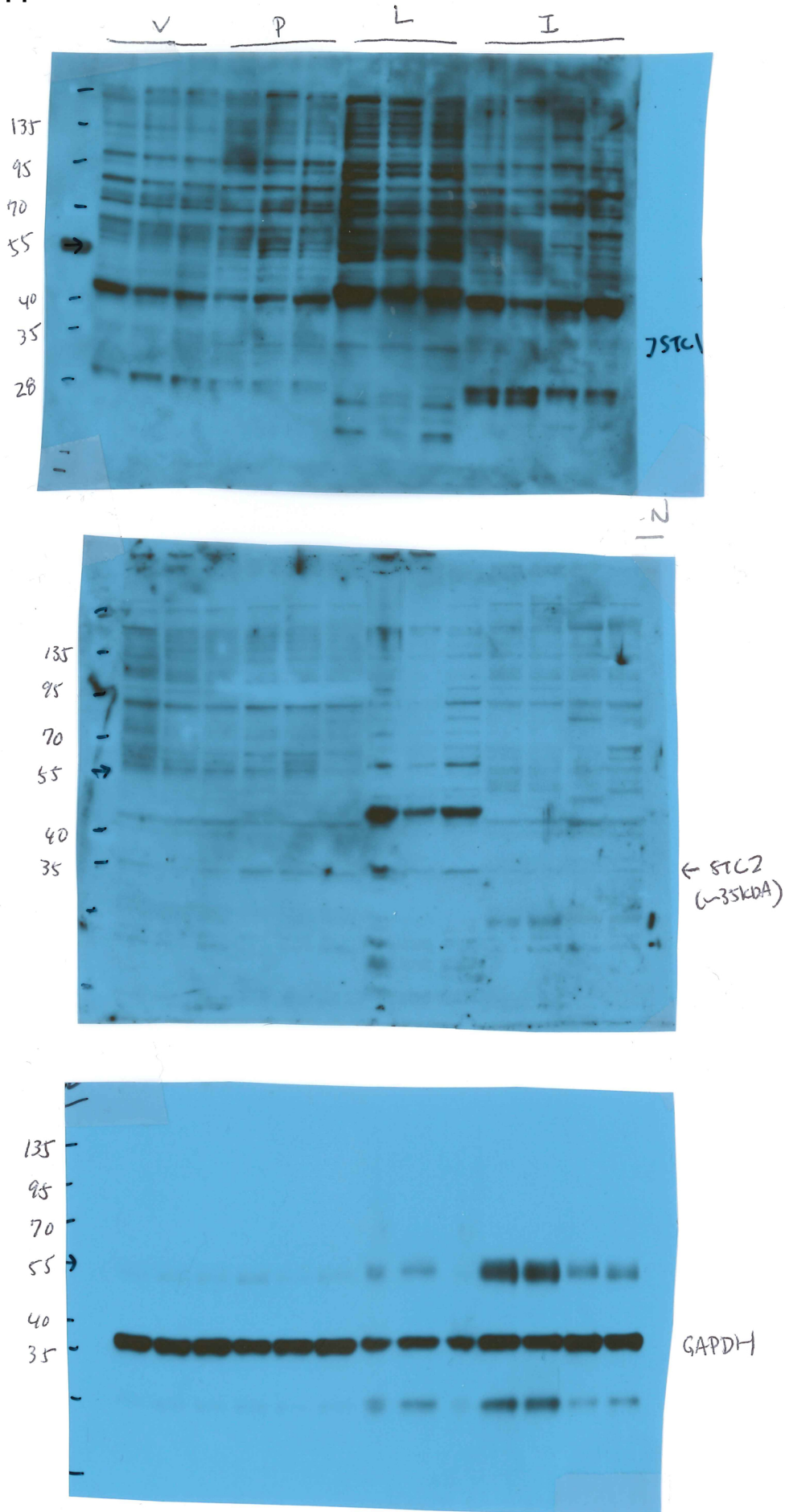

Supplement: Supplementary file 7 — Source Data for Figure 6 [file EMMM-8-388-s005.pdf]
